# Scale-down characterization of post-centrifuge flocculation processes for high-throughput process development

**DOI:** 10.1002/bit.25313

**Published:** 2014-09-02

**Authors:** Georgina Espuny Garcia del Real, Jim Davies, Daniel G Bracewell

**Affiliations:** 1 The Advanced Centre for Biochemical Engineering, Department of Biochemical Engineering, University College LondonTorrington Place, London, WC1E 7JE, UK; 2 Lonza Biologics plcSlough, Berkshire, SL1 4DX, UK

**Keywords:** primary separation, flocculation, high-throughput, ultra scale-down, scale-up, micromixing

## Abstract

Flocculation unit operations are being revisited as a strategy to ease the burden posed on clarification and purification operations by the increasingly high cell density cultures used in the biopharmaceutical industry. The purpose of this study was to determine the key process parameters impacting flocculation scale-up and use this understanding to develop an automated ultra-scale down (USD) method for the rapid characterization of flocculation at the microliter scale. The conditions under which flocculation performance of a non-geometrically similar vessel three orders of magnitude larger can be mimicked by the USD platform are reported. *Saccharomyces cerevisiae* clarified homogenate was flocculated with poly(ethyleneimine) (PEI) to remove the residual solids remaining in the centrate. Flocculant addition time modulated flocculation performance depending on the predominant mixing time scale (i.e. macro-, meso- or micromixing). Particle growth and breakage was mimicked at the two flocculation scales by the average turbulent energy dissipation (ε_avg_) and impeller tip speed (v_tip_) scale-up bases. The results obtained were used to develop an USD method. The USD method proposed uses constant ε_avg_ as the scale-up basis under a micromixing controlled regime. These conditions mimicked the STR flocculation performance within a ±5% error margin. Operation in the mesomixing regime led to particle size deviations between the flocculation scales of ≤50 %. These results, in addition to the microscopic observations made, demonstrate the USD system presented in this work can produce process-relevant flocculated material at the microliter scale under the correct operating conditions.

## Introduction

Polyelectrolyte flocculation has been implemented in the wastewater treatment industry for at least four decades (Bolto and Gregory, 2007; Renault et al., 2009), but more recently its use has been proposed for biopharmaceutical processes (Riske et al., 2007). The clinical and commercial success of biologics has led to the need for a large global manufacturing capacity (Anicetti, 2009). This demand has seen significant improvements in upstream productivity; however, this has placed increased requirements on primary recovery. New clarification technologies capable of coping with increased levels of cell debris and other impurities normally associated with increased cell densities are now needed (Westoby et al., 2011). It is in these circumstances that flocculation is being revisited as a less expensive approach that at the same time it may also offer economies of scale (Low et al., 2007).

One of the earliest studies in which polyelectrolyte flocculation was used to partially clarify biological feeds was published more than 20 years ago (Bonnerjea et al., 1988). The flocculation mechanisms and the purification opportunities of polyelectrolyte-induced flocculation were initially investigated using *Saccharomyces cerevisiae* and its homogenate (Cordes et al., 1990; Milburn et al., 1990; Salt et al., 1995, 1996). Flocculation studies on *Escherichia coli* feeds and others from bacteria followed. Barany and Szepesszentgyörgyi (2004), Shan et al. (1996) and Strand et al. (2003) looked into gaining more understanding of the key variables affecting flocculation performance. Finally, flocculation of mammalian cells was evaluated for the removal of cells and process-related impurities in combination with established technologies such as depth filtration (Singh et al., 2013) or centrifugation followed by depth filtration (Riske et al., 2007).

Flocculation agents are classified into inorganic and polymeric materials. Polymers are regarded as more effective at a comparatively lower concentration, from mechanically stronger flocs and are efficient over a wide range of pH and temperatures. Polymeric flocculants are further divided into natural (e.g. starch or cellulose) and synthetic materials, and into cationic, anionic or non-ionic depending on their electrostatic nature. Positively charged synthetic polymers are preferred for biomedical and pharmaceutical applications since they are chemically synthesized and more efficient in removing the generally negatively charged bio-colloids. Nonetheless, their use in this industry demands that manufacturers comply with current Good Manufacturing Practice (cGMP) to minimize the problems associated with the high variability of synthetic polyelectrolytes (i.e. residual unreacted monomers or other chemicals). Concerns about possible toxicity effects, removal difficulties and their final cost at large manufacturing scale also arise (Aunins and Wang, 1989; Renault et al., 2009; Thömmes and Etzel, 2007).

The success of flocculation unit operations is determined by a large number of variables and factors (Bratby, 2006; Kim et al., 2001; Salt et al., 1995). Such large experimental space implies the need for high-throughput, automation and multi-factorial design approaches during the initial process development studies. Nonetheless, no attempts have been made to develop a flocculation platform that (i) generates process-relevant feed material at the microliter scale; and (ii) obtains data equivalent to that of production scale. An USD flocculation technology is necessary to explore the large experimental space and to better understand the key process interactions between flocculation and the subsequent unit operations, e.g. centrifugation (Berrill et al., 2008), microfiltration (Kim et al., 2001), depth filtration (Singh et al., 2013) or centrifugation followed by depth filtration (Riske et al., 2007). Such technologies are required to generate the process understanding early in process development so that time to market, risk of failure and associated costs are reduced (Titchener-Hooker et al., 2008). The USD approach has been successfully established for centrifugation (Tait et al., 2009), normal flow filtration (Jackson et al., 2006; Kong et al., 2010) and chromatography (Wenger et al., 2007; Wiendahl et al., 2008), but it is yet to be proved for other unit operations such as flocculation.

This study is focused on developing an USD platform for pre-clarified cell broths in which the optimal conditions for the flocculation and removal of solids remaining in the centrate can be studied. A multiwell agitated system that could be fitted on the deck of a liquid handling robot was employed. The system contained a magnetically-driven rotating disc impeller in each well which could be operated continuously and independently from the liquid arm used for controlled reagent additions into the wells. This mixing system was selected for its ability to handle solids and viscous polymeric flocculants as well as resembling the mechanics of a conventional impeller (when compared to shaking systems). *Saccharomyces cerevisiae* was chosen to establish this USD platform and PEI was the flocculant of choice. Flocculation was carried out on clarified homogenate in order to examine its use to remove micronized debris and colloids in early-purification. Emphasis on distinguishing between flocculation and polyelectrolyte-induced precipitation is given. In this article the term ‘polyelectrolyte flocculation’ describes the destabilization of a biocolloidal suspension upon addition of a cationic polymer causing the particulates to agglomerate giving rise to flocs. Flocculation is understood to act on particles that remain stable as separate entities in solution (i.e. colloidal suspensions) (Bratby, 1980) and not on soluble molecules such as proteins coming out of solution in the presence of a specific polymer and upon changing the bulk parameters of the medium (Capito et al., 2013; McDonald et al., 2009; Peram et al., 2010).

**Figure 1:**
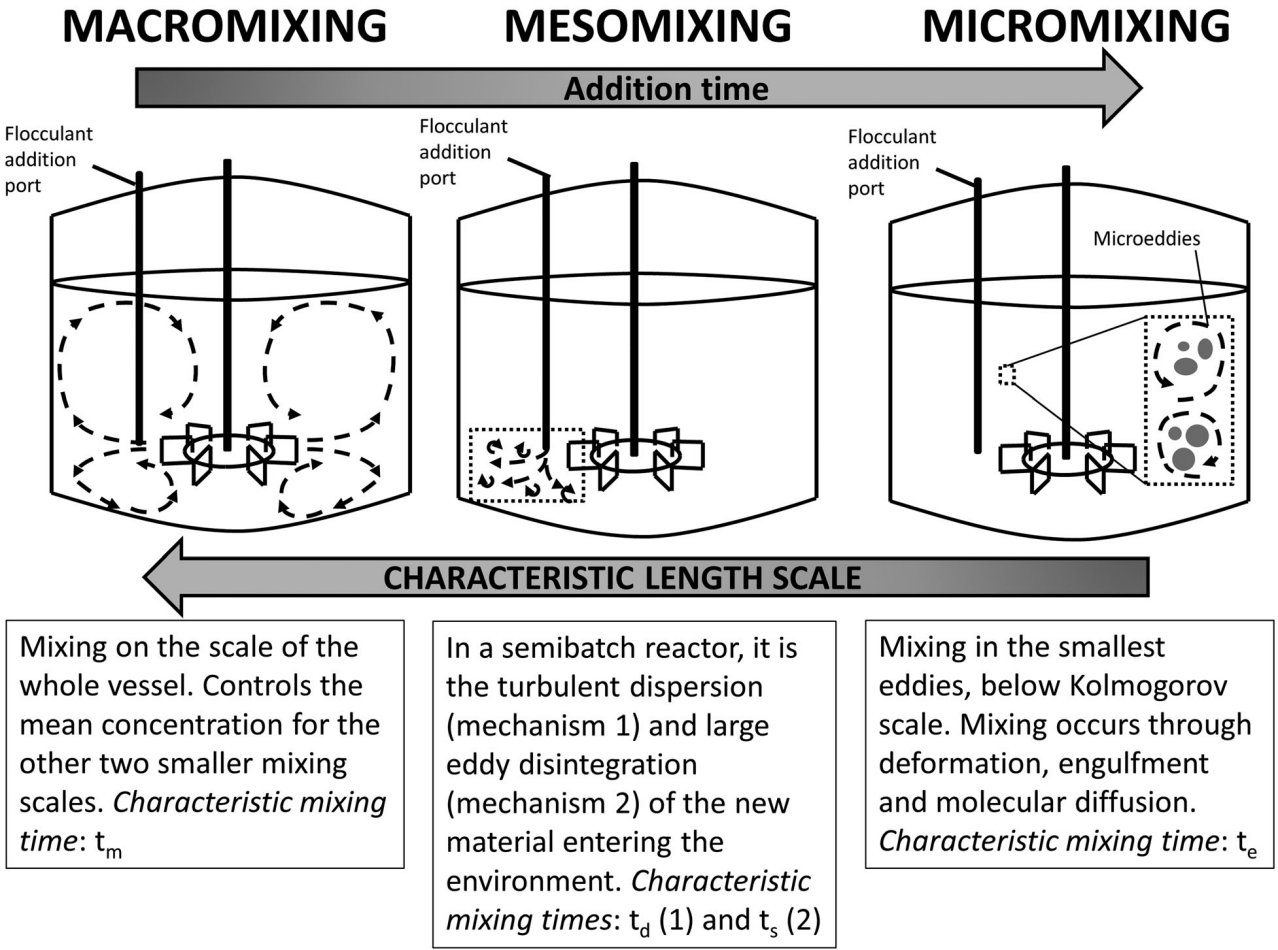
Schematic diagram of the characteristic time and length scales of mixing in a standard STR equipped with 6-bladed disk turbine (Baldyga and Bourne, 1992, 1999; Baldyga and Pohorecki, 1995).

The objective of this article was to investigate the key parameters dominating during flocculation scale-up/scale-down and develop an USD flocculation platform based upon this. The approach was to keep all but the scale-dependent flocculation variables constant, e.g., flocculant addition time and impeller speed. Flocculant addition time was scaled-up by determining its effect on the predominant mixing time scale (whether macromixing, mesomixing or micromixing; see Fig. 1) and matching it at both flocculation scales. The second variable, impeller speed, was scaled by evaluating and selecting scale-up correlations from the literature.

## Theoretical Considerations

### Mixing Time Scales and Their Characterization

Mixing time scales become important when the kinetics of a reaction are faster or are of the same order as the mixing process (Shaer et al., 1999). There are three main mechanisms according to the scale of the vessel at which they are relevant (i.e. characteristic length scale) (Baldyga and Bourne, 1992, 1999; Baldyga and Pohorecki, 1995; Vicum et al., 2004):

*Macromixing*. For a baffled tank and fully developed turbulence, its characteristic mixing time is approximated as follows:

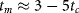
(1)where *t*_*m*_ is the macromixing time (s) and *t*_*c*_ is the circulation time (s). For a six-bladed disk turbine, *t*_*c*_ is calculated as below:

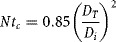
(2)where *N* is the impeller speed (s^−1^), *D*_*T*_ is the vessel diameter (m) and *D*_*i*_ is the impeller diameter (m).

*Mesomixing* occurs via two specific mechanisms: (i) turbulent dispersion; and (ii) large eddy disintegration. Assuming a stationary, homogeneous and isotropic turbulent environment the turbulent dispersion characteristic time is calculated as:

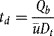
(3)where *t*_*d*_ is the dispersive mesomixing time (s), *Q*_*b*_ is the feed rate (m^3^.s^−1^), 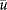 is the fluid velocity in the vicinity of the feed pipe (m.s^−1^) and *D*_*t*_ is the turbulent diffusivity (m^2^.s^−1^). Under the same assumptions, large eddy disintegration characteristic time is approximated as:

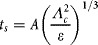
(4)where *t*_*s*_ is the characteristic time for large eddy disintegration mesomixing (s), *A* is a constant in the range of 1–2, *Λ*_*c*_ is the macroscale concentration (m) and *ε* is the local turbulent energy dissipation per unit mass (W.kg^−1^).

*Micromixing* characteristic time is calculated as follows:

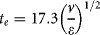
(5)where *t*_*e*_ is the micromixing time (s) and ν is the kinematic viscosity (m^2^.s^−1^).

Micromixing can be also characterized experimentally by physical and chemical methods (Fournier et al., 1996). The system of parallel competing reactions developed by Fournier et al. (1996) was chosen for this work. The reader is referred to Guichardon and Falk (2000) for further details about this method and its experimental procedure.

Literature reports that increases in viscosity affect micromixing (Baldyga and Bourne, 1999; Bourne et al., 1989, 1995; Gholap et al., 1994). Aqueous solutions are used for the chemical characterization of micromixing, thus viscosity values are close to 1. It is therefore necessary to study the viscosity of the solutions to be flocculated to screen for possible significant increments.

### Scale–up Correlations in STRs for Turbulent Conditions

The estimation of the distribution of ε in the flocculation systems presented was not pursued in this article despite its use to describe the hydrodynamic stresses encountered in the vessel (Henzler, 2000; Hortsch and Weuster-Botz, 2010; Zhou and Kresta, 1996). Instead, the aim was to assess the use of established “rules of thumb” using average turbulent energy dissipation and impeller tip speed, which have been traditionally used to size mixing vessels:

1. Average turbulent energy dissipation
  Constant power input per unit volume has been used as a scale-up basis with considerable success in some areas (Chester and Oldshue, 1987; Kresta, 1998; Uhl and Von Essen, 1986). This ratio is also described as the average energy dissipation per unit mass in a vessel (Hortsch and Weuster-Botz, 2010):

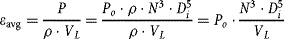
(6)where *ε*_avg_ is the average turbulent energy dissipation per unit mass (W.kg^−1^), *P* is the power input (W), *ρ* is the liquid density (kg.m^−3^), *V*_*L*_ is the liquid volume (m^3^) and *P*_*o*_ is the impeller power number. For the pilot STR six-bladed disk turbine *P*_*o*_ = 5.8 (McCabe et al., 2005) and for the USD magnetically-driven disc impeller *P*_*o*_ = 0.86 (value empirically estimated from a global best fit).
  Constant *N*^3^*D*_*i*_^2^ and constant average shear rate (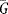) are two other scale-up correlations which are proportional to *ε*_avg_. Therefore, this paper only considered constant *ε*_avg_ to investigate the use of constant *P*/*V*_*L*_ as a scale-up basis between the flocculators described.
2. Impeller tip speed
  A second scale-up correlation is constant torque intensity or torque per unit volume (T/V_L_), which corresponds to constant peripheral velocity or impeller tip speed (Chester and Oldshue, 1987; Uhl and Von Essen, 1986). This is calculated as follows (Doran, 1995):

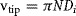
(7)where v_tip_ is the impeller tip speed (m.s^−1^).

These rules imply partial geometric similarity between the systems. However, performance predictions can be performed if the key process variables dominating when scale changes are made are analyzed (Titchener-Hooker et al., 2008; Zlokarnik, 2002); in this article this is achieved by means of studying impeller speed and flocculant addition time.

## Materials and Methods

### Feed Preparation

All chemicals used in this study were of AnalaR (or equivalent) grade and, unless otherwise stated, purchased from Sigma Ltd. (Gillingham, UK). Clarified yeast homogenate was prepared as follows: high activity Baker’s yeast (*Saccharomyces cerevisiae*) provided by DCL London (London, UK) was suspended to 28% packed wet weight per volume in phosphate buffer (0.1 M NaH_2_PO_4_, adjusted to pH 6.5 using 3 M NaOH), then homogenized by performing five discrete passes at 500 bar pressure through a Lab60 continuous flow high-pressure homogenizer (APV UK Ltd., Crawley, UK) and finally clarified by centrifugation (45 min at 6,300 rpm in a Beckam Avanti J-E centrifuge fitted with a JA-10 rotor - Beckam Coulter Ltd., High Wycombe, UK). The supernatant was recovered and stored at −20°C for future use. The clarified yeast homogenate had a final protein concentration of 30 ± 3 g.L^−1^ (Pierce™ BCA protein assay, Thermo Scientific, Rockford, IL), liquid density equal to 1.02 kg.L^−1^ and kinematic viscosity equal to1.63cSt at 22°C (Brookfiled DV-II+ viscometer, Brookfield Engineering Laboratories Inc., MA).

### Flocculation Systems Configuration

**Figure 2:**
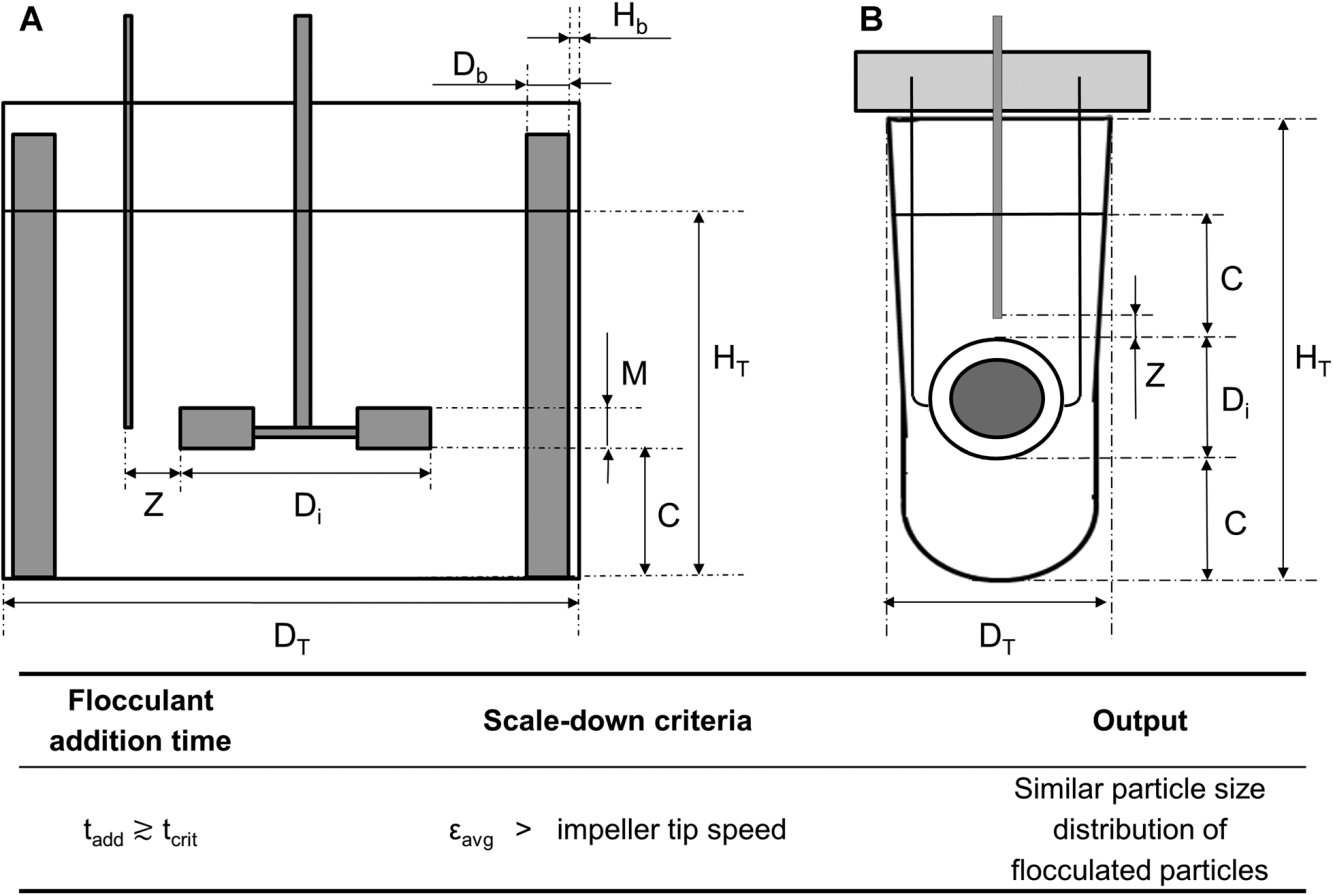
Diagram of the pilot scale (A) and ultra scale-down (B) flocculation systems. Key geometrical ratios and dimensional specifications are as follows: A. H_T_:D_T_ = 1:1; D_i_:D_T_ = 1:3; D_i_:C = 1:1; D_T_:D_b_ = 1:10; D_T_:H_b_ = 1:100; M:D_i_ = 1:5; Z = 13 mm. B. D_T_ = 8.4 mm; D_i_ = 5.5 mm; C = 5.0 mm; H_T_ = 21 mm; Z = 1 mm. Note diagram is not to scale.

*Pilot scale STR* - see Figure 2A for diagram. This was a 2L vessel of 125 mm diameter (D_T_) and 1.5 L working volume (V_L_), equipped with a 41.5 mm diameter (D_i_) six-bladed disk turbine. Reagent additions were via a 3.0 mm internal diameter pipe positioned 13 mm away from the center of the impeller blade and controlled with a syringe pump (Ultra programmable PHD Ultra, Harvard Apparatus Ltd., Kent, UK). *USD 96-well plate* – see Figure 2B for a diagram of a single well of the plate. The impeller was a parylene encapsulated magnetic disc (V&P Scientific, San Diego) mounted on a perforated Perspex lid through a fixed Teflon seal. The microplate was a standard storage plate (1.2 mL square round-bottom plate, ABGene Ltd, Epsom, UK) with 800 µL working volume per well located on a magnetic stirrer (710CI, V&P Scientific) and mounted on the deck of a liquid handling robot (Evo 150, Tecan UK Ltd, Reading, UK). A calibrated stroboscope was used to confirm the coupling between magnetic discs and the magnetic drive. Reagent additions were via stainless steel tips of 1 mm internal diameter located in the center of the microwell with the outlet 1.0 mm above the disc tip.

### Calculation of Mixing Time Constants

Mixing time constants for pilot scale were calculated for an impeller speed of 4.8 rps as described in the Section Theoretical Considerations. The value of 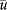 was approximated by the mean radial and mean tangential velocities at a radial coordinate equal to the distance from the vessel vertical axis to the feed point determined by Wu and Patterson (1989). The values of *D*_*t*_ and *Λ*_*c*_ were calculated according to Baldyga and Bourne (1999). The *ε*/*ε*_avg_ correlation for region 2 of the experimental flow model described by Bourne and Yu (1994) was used to calculate the local turbulent energy dissipation rate.

### Micromixing Time Studies

Critical addition time studies were carried out using the iodide-iodate system of parallel competing reactions as described by Guichardon and Falk (2000). A solution of 0.5 M sulphuric acid was used (98 % v/v concentrate, Merck KGaA, Darmstadt, Germany). Total acid volume injected equaled to 8 × 10^−3^ L the system’s working volume. The bulk solution contained the following: [KIO_3_] = 2.33 × 10^−3^ M, [KI] = 11.6 × 10^−3^ M, [H_3_BO_3_] = 182 × 10^−3^ M and [NaOH] = 90.9 × 10^−3^ M (VWR International, BDH Prolabo, Leuven, Belgium). Critical addition times (t_crit_) were estimated at 1.8, 4.8, and 7.9 rps (STR) and 17 and 27 rps (microwell); these values fall within the range of impeller speeds studied in this work. Acid addition time (t_add_) ranged from 340 s to 3.8 s (STR) and from 6.4 s to 7.0 × 10^−3^ s (microwell). The increments in viscosity caused by the flocculant and clarified homogenate solutions were found to be modest in comparison to the published literature values where significant changes in micromixing were found (Baldyga and Bourne, 1999; Bourne et al., 1989, 1995; Gholap et al., 1994). Thus, the viscosity of the above aqueous solutions was not increased with a viscosity-raising additive.

### Flocculation Studies

Flocculation was performed at pH 6.5 by addinga 4% w/v PEI stock solution (kinematic viscosity equal to 1.92 cSt at 22°C; Brookfiled DV-II+ viscometer, Brookfield Engineering Laboratories Inc., MA) to a final concentration of 0.2% v/v and a maximum feed dilution of 5.0% v/v. An aging time of 600 s was allowed for the floc to maturate (shorter ageing times have successfully flocculated similar feeds with PEI; Milburn et al., 1990). In microwells flocculant addition rates varied from 1 to 900 µL.s^−1^ when using fixed impeller speeds (17 rps or 20 rps) or a fixed flocculant addition rate (1 µL.s^−1^) was used with varying impeller speeds (from 5.3 to 27 rps). Flocculation was performed in 4 different wells and used for multiple particle size analysis. At pilot scale flocculant addition rates ranged from 15 to 184 mL.min^−1^ when using a fixed impeller speed of 4.8 rps, or a fixed addition rate (15 mL.min^−1^) was used at impeller speeds varying from 1.8 to 7.9 rps.

### Particle Size and Image Analysis

Low angle laser light diffraction (Mastersizer 2000 connected to a Hydro 2000SM wet dispersion unit, Malvern Instruments Ltd., Malvern, UK) was used for particle size analysis. The flocculated feed was dispersed in 0.22 µm filtered ultrapure water (Millipore, UK) to a red laser obscuration of 13–19 %. Three particle size measurements of the same sample were consecutively taken over a period of time to ensure no particle breakage was occurring in the dispersion unit. Phase contrast images of the flocculated particles were taken with a Nikon TE2000-PFS inverted microscope (Nikon Instruments Europe B.V, Badhoevedorp, The Netherlands) equipped with a charge-couple device camera. Process image analysis was then carried out with ‘ImageJ v. 1.47’ (http://rsb.info.nih.gov/ij/).

## Results and Discussion

### The Effect of Mixing Time Scales on PSD

The initial aim was to investigate how mixing time scales modulated the flocculation process in the pilot and microwell systems. This was performed by varying the flocculant addition time (t_add_). Flocculation performance was described by the particle size characteristic descriptors d_10_, d_50_, and d_90_ as well as by PSD curves in an attempt to reveal complex populations (i.e. bimodal or tri-modal distributions) that the values of d_10_, d_50_ and d_90_ could not.

**Figure 3:**
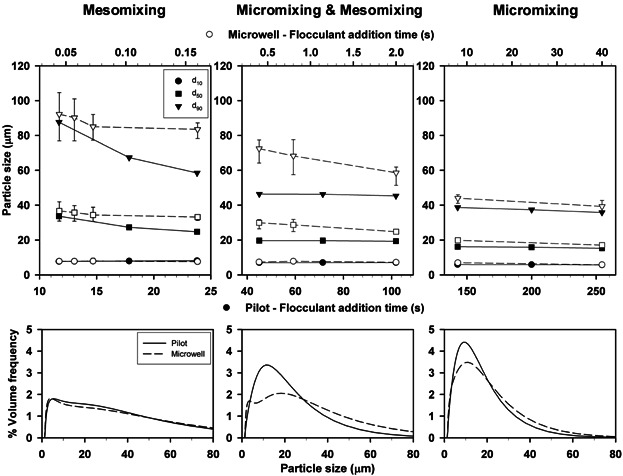
Effect of t_add_ on PSD in the microwell and pilot scale flocculation systems. Impeller speed was kept constant at 17 rps (microwell) and 4.8 rps (pilot). Error bars describe range of values where n = 8. PSD curves (B) were obtained at 184, 47.6 and 8.6 mL.min^−1^ (pilot) and 900, 50 and 1 µL.s^−1^ (microwell) flocculant addition rates.

Figure 3A shows that wider floc distributions were gradually obtained with decreasing flocculant addition time in both flocculation scales. However, the spread in the PSD differed depending on the flocculation system and the predominant mixing time scale. At pilot scale, the characteristic time constants were calculated by applying Equation 2009 to Equation 1999 to determine which was the predominant mixing time scale in each of the t_add_ studied. The results are summarized in Table 1. In the STR, macromixing was thought not to be limiting at the range of t_add_ studied (i.e. t_add_ and t_m_ and t_c_ were not of similar magnitude; Baldyga et al., 1993; Baldyga and Bourne, 1999) and t_m_ remained unaffected by the changes in t_add_. Therefore, micromixing and mesomixing were the mixing time scales controlling the PSD of the flocs formed at pilot scale. Micromixing governed at low flocculant addition rates (i.e. t_add_ > 140 s). Across the micromixing regime constant values of d_10_ and d_50_ were found, while the value of d_90_ increased by 8%. A step increase in the values of d_50_ and d_90_ took place when t_add_ was in the 102 s to 45 s range, which corresponded to increments of 4 µm and 9 µm respectively. These larger PSDs may be explained by both micromixing and mesomixing time scales influencing flocculation despite the micromixing time predictions continuing to be larger than those expected for mesomixing (i.e. t_e_ > t_d_ and t_s_). This situation changed at t_add_ < 24 s, when mesomixing was the only controlling mechanism (i.e. t_d_ > t_e_). At the USD scale the PSD of the flocs generated gradually decreased with longer t_add_. Equation 2009–Equation 1999 could not be applied and the mixing time scales were therefore experimentally characterized (see Section Experimental Characterization of Micromixing).

**Table 1:** The mixing time constants derived from the equations in section 2.1 for the pilot scale STR flocculant addition time studies (see Nomenclature for the definition of the terms)

	t_add_ (s)	t_c_ (s)	t_m_ (s)	t_d_ (s)	t_s_ (s)	t_e_ (s)
**Micromixing**	254			0.012	0.014	
	200	1.6	6.4	0.015	0.015	0.081
	143			0.021	0.017	
**Micromixing**	102			0.030	0.019	
**&**	71	1.6	6.4	0.042	0.022	0.081
**Mesomixing**	45			0.067	0.025	
**Mesomixing**	24			0.127	0.031	
	18	1.6	6.4	0.170	0.034	0.081
	12			0.259	0.040	

Figure 3B shows the PSD curves across the three mixing regimes. Under mesomixing controlling conditions comparable PSD curves (variation <10 % between 3–70 µm) were obtained at both flocculation scales despite the fact that impeller speed and t_add_ differed in the two flocculation systems. These results are explained by the relatively fast flocculant addition rates used causing flooding of the impeller region by an excess concentration of the polymer. Bolto and Gregory (2007) reported the importance of an even distribution of the added flocculant throughout the flocculation vessel to avoid local excess flocculant concentrations as these lead to non-uniform adsorption and re-stabilization of the colloidal suspensions. When both micromixing and mesomixing modulated the flocculation performance, monomodal PSDs were obtained at pilot scale while bimodal and wider PSDs were observed at USD scale. Finally, narrower monomodal distributions with a maximum difference of 14% in the values of d_10_, d_50_, and d_90_ were observed at both flocculation scales under a micromixing controlled regime. Pre-treated feeds with narrow monomodal PSDs are always of interest, particularly if they are to be subsequently processed by centrifugation (Berrill et al., 2008) or depth filtration (Singh et al., 2013).

**Figure 4:**
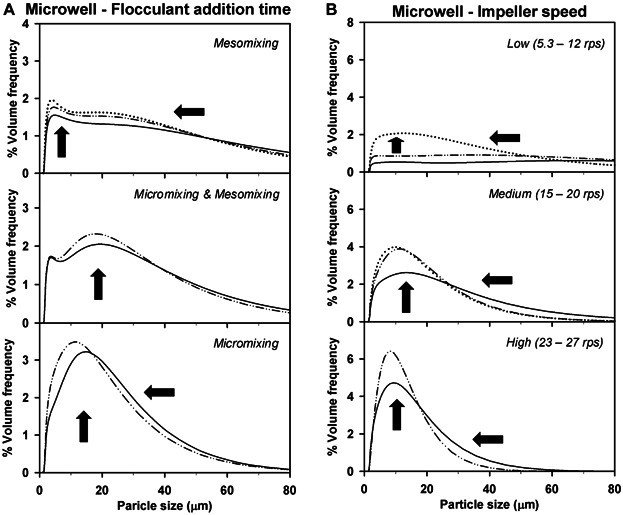
Effect of flocculant addition time (A) and impeller speed (B) on flocculation performance for the microwell system. Arrows show the shift in particle size distribution with longer addition times (A) or faster impeller speeds (B). Flocculation experiments were carried out at fixed impeller speed (17 rps) with varying flocculant addition rates (1–900 µL.s^−1^) (A) and at varying impeller speeds (5.3–27 rps) with fixed flocculant addition rate (1µL.s^−1^) (B). Mixing scales (A) were assigned as in Figure 3.

At each flocculation scale the complexity of the PSDs obtained was also determined by the predominant mixing time scale. Figure 4A describes that in the USD system longer t_add_ shifted bimodal and dispersed populations to monomodal and narrower distributions. This trend, also observed at pilot scale (data not shown), is explained by long t_add_ allowing a more homogeneous distribution of the polyelectrolyte inside the vessel. Similar results were reported by Berrill et al. (2008). They described that highly variable PSDs were obtained when using high flocculant addition rates on *Escherichia coli* feeds.

### Experimental Characterization of Micromixing

The results obtained in Section The Effect of Mixing Time Scales on PSD showed the need to characterize the mixing time scales at USD scale. The iodide-iodate reaction system was used to study the segregation state of the fluid in the microwell flocculation system by the changes in the value of the segregation index (X_S_) with increasing t_add_. The STR was also characterized using the same method for comparison purposes.

**Figure 5:**
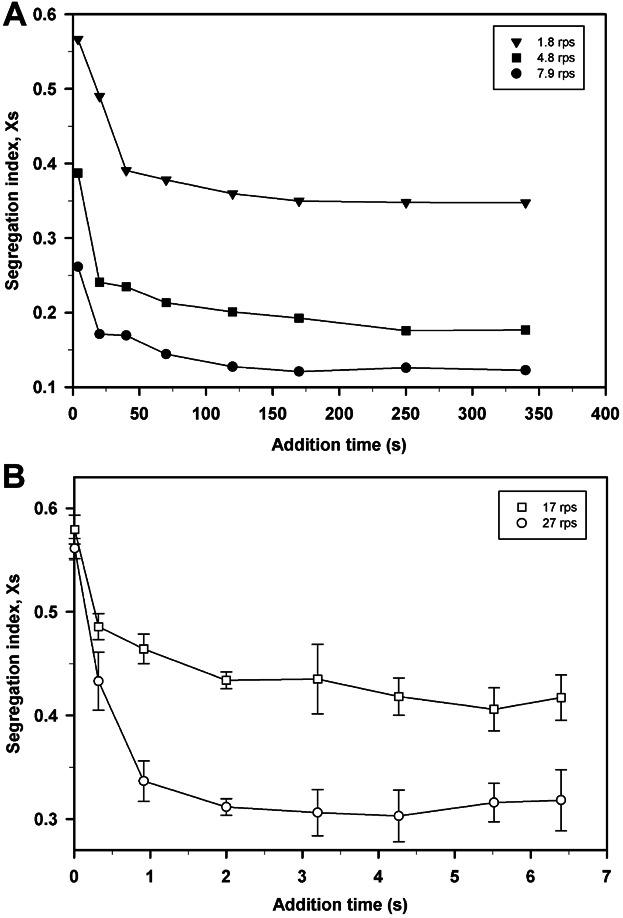
Micromixing time studies for the pilot scale (A) and microwell (B) flocculation systems performed at different values of impeller speed. Error bars describe one standard deviation where n = 8.

Figure 5A describes how the value of X_S_ gradually decreased with increasing t_add_ for the three impeller speeds studied at pilot scale. This trend indicated improved micromixing with longer t_add_, which was also achieved at higher values of impeller speed. The transition from a micromixing and mesomixing controlled regime to micromixing only (Baldyga and Bourne, 1992) was indicated by the plateauing in the value of X_S_ at t_add_ ≈ 150 s. This transition, identified as the critical addition time (t_crit_) for a particular vessel and fixed hydrodynamic conditions, occurred at the three impeller speeds studied. The value of t_crit_ corroborated the theoretical approximation of the time constants presented in Table 1. At USD scale (Fig. 5B) the same trends of X_S_ over t_add_ and increasing values of impeller speed were observed. Critical addition time occurred at t_add_ ≈ 3 s hence indicating the system was only micromixing controlled at t_add_ > 3 s. In this flocculation system the influence of macromixing could not be determined theoretically or experimentally. In these cases, if the predominance of the micromixing scale wants to be guaranteed a larger value of t_add_ than the t_crit_ experimentally obtained needs to be chosen; the effects of macromixing and mesomixing can be neglected if sufficiently slow additions are used (Assirelli et al., 2002; Baldyga and Bourne, 1999).

For this application macromixing and mesomixing regimes were not of interest as they resulted in the creation of wide PSDs challenging for solid-liquid separation steps (see Fig. 3) and they require rapid flocculant addition rates which were unsuited for scale-up. Therefore, to assure a micromixing controlled regime the subsequent flocculation experiments were carried out at values of t_add_ = 152 s for the pilot and t_add_ = 40 s for the USD flocculation systems.

Different values of X_S_ were obtained at both flocculation scales since they are associated with the location of the feed point and the reaction zone (Baldyga and Bourne, 1999) as well as vessel geometry. These results evidence the need to characterize the changes in the value of X_S_ with increasing t_add_ in each mixing system studied. Backmixing effects in the micromixing regime were not seen despite the long addition times used as no increase in the value of X_S_ was observed with longer t_add_ (Baldyga et al., 1993). Figure 5 then confirms that the feed points were appropriately located and oriented relative to the fluid flow pattern in the vessel.

### Flocculation Scale-up Correlations

**Figure 6:**
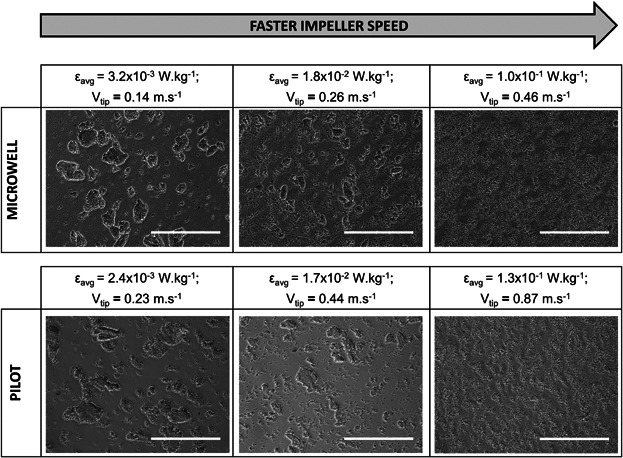
Representative images of flocculated samples obtained at increasing values of impeller speed in the microwell and pilot scale flocculation systems. Bar size indicates 200 µm. The values of ε_avg_ and v_tip_ for the microwell and pilot-scale systems at which the flocs were generated are specified above each corresponding picture.

Using the selected micromixing controlled region scale-up correlations from the literature were evaluated to find one to allow the microwell system to be predictive of flocculation performance of a larger non-geometrically similar STR vessel. Impeller tip speed and average turbulent energy dissipation were selected as established parameters for impeller based mixing systems (see section 2.2). Flocculation experiments using the pilot and microwell systems at varying impeller speeds were performed and their PSD studied. In accordance with the results obtained by Shamlou et al. (1996), particle growth and breakage depended upon the hydrodynamic conditions inside the vessel but were independent of the source providing the liquid motion. Increasing values of impeller speed resulted in narrower PSDs both in the microwell (Fig. 4B) and the pilot scale flocculation systems (data not shown). The shift of the monomodal PSDs, which confirmed the predominance of a micromixing regime, to progressively smaller sizes with increasing values of impeller speed indicated particle fragmentation. Particle break-up resulted in small particles increasing their values of percentage volume frequency. The rapid decrease in particle size and increase in the percentage volume frequency of small particles seen between 5.3 and 20 rps indicated that particles formed under low shear environments were more susceptible to breakage than those exposed to high impeller speed values. The same impeller speed effect on floc size could be observed microscopically (Fig. 6), which also confirmed that comparable particles were obtained in both flocculation systems under similar values of ε_avg_ or values of v_tip_ that differed two fold.

**Figure 7:**
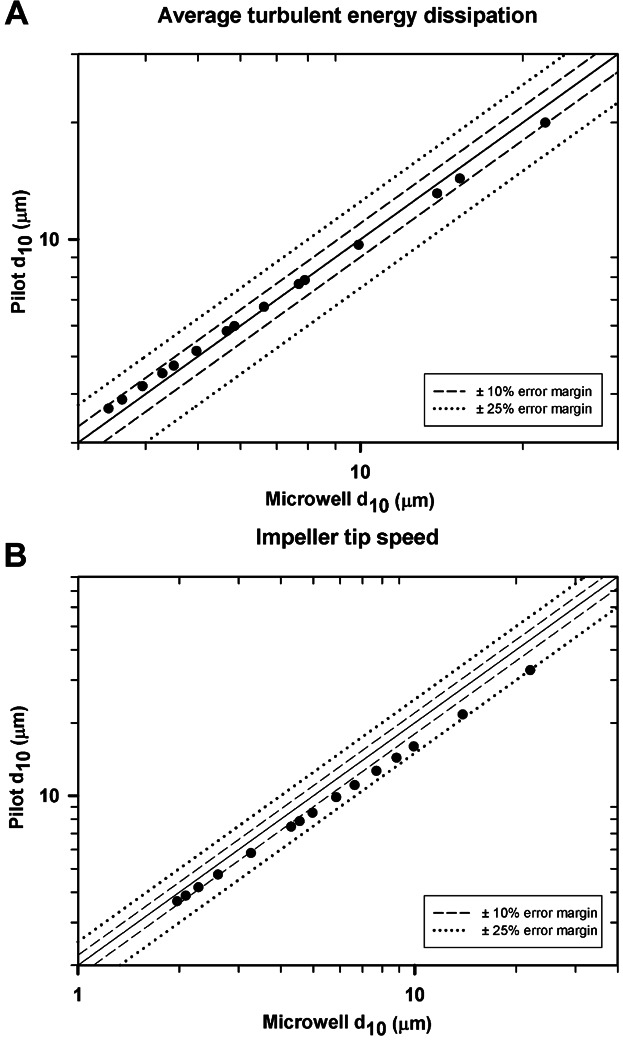
Parity plots of the predicted pilot scale and microwell d_10_ data size range. Graphs describe the correlation between the two flocculation systems when using ε_avg_ (A) and v_tip_ (B) as the scale-up basis. The x-axis of the v_tip_ plot was adjusted relative to the y-axis by a factor of 2. Each data point corresponds to a different value of impeller speed used in the flocculation studies.

Using d_10_, d_50_ and d_90_ data sets and plotting against ε_avg_ or v_tip_, power correlations within R^2^ ≥ 0.95 were established. These were used to generate parity plots which considered the range of impeller speeds studied. Figure 7 shows the parity plots for the d_10_ size range. This size range is prioritized since it represents the material most difficult to separate and that which flocculation should target.

The data followed a one to one correlation when using ε_avg_ (Fig. 7A), but when scaling up based on constant v_tip_ (Fig. 7B) a global best fit was achieved when the STR was operated at twice the USD impeller tip speed. The d_10_ data set was predicted within a ±10% error margin by the ε_avg_ scale-up correlation. When scaling based on v_tip_ the margin increased to ±25% since the larger flocs generated under a transitional flow regime (Reynolds number or Re from 3 × 10^3^ to 10^4^; Doran, 1995) were not tightly correlated. In the d_50_ data sets (data not shown), the particles formed under a transitional flow regime laid within the ±10% error margin line irrespective of the scale-up criterion used; under a turbulent regime (Re > 10^4^; Bates et al., 1966) they laid beyond this line. With an error margin of ±25% the same results were observed for the d_90_ data sets (data not shown). Both correlations proved to be successful scale-up bases but ε_avg_ was chosen for further flocculation studies for its ability to more accurately predict the PSD of small flocs. In a polydisperse population the smallest particles determine the filter pore size in depth filtration and microfiltration unit operations as well as the supernatant clarification in centrifugation processes.

Average turbulent energy dissipation averages the widely differing ε values occurring in the vessel and can therefore underestimate the impact that the high values of ε present in the impeller region have upon the flocculated particles (Zhou and Kresta, 1996). Nonetheless, in the case of isotropic turbulence and when micromixing determines the process output, P/V_L_ is the pertinent scale-up criterion (Bourne and Yu, 1994; Zlokarnik, 2002). Micromixing was assured by performing the flocculation studies at sufficiently long t_add_. However, the condition of isotropic turbulence was achieved differently in each flocculation system. At USD scale, the oversized impeller guaranteed isotropic turbulence inside the well since increasing values of D_i_/D_T_ decrease the values of maximum energy dissipation per unit mass (ε_max_) encountered in the reactor (Henzler, 2000; Hortsch and Weuster-Botz, 2010). At pilot scale the flocculation studies were carried out at Reynolds values below a fully developed turbulent regime (i.e. Re ≤ 1.3 × 10^4^); therefore, the ε differences between the impeller area, where the ε values are expected to be at least 10 times larger than in the bulk of the tank (Zhou and Kresta, 1996), and the rest of the vessel are thought to be reduced. This hypothesis, which invalidates the assumptions of fully turbulent regimes and local isotropy for ε_avg_, is reinforced by the deviations observed in the d_50_ and d_90_ data sets for turbulent conditions at pilot scale.

The close correlation between pilot and microwell PSDs shown in Figure 7B is explained by v_tip_ determining maximum fluid shear rate or shear stress (Uhl and Von Essen, 1986). The global best fit between the v_tip_ of the pilot STR flocculation system used and the USD system needs to be determined *a priori*. This represents an additional characterization step during the early process development studies. Hence ε_avg_ was selected because it can predict the d_10_ particle size range without the need to previously characterize the flocculation systems in use.

### Validation of the Flocculation Scale-Down Methodology

In order to validate the use of ε_avg_ as a scale-up basis, flocculation studies were performed using the same value of ε_avg_ at both flocculation scales and at a range of t_add_ so that the three mixing time scales were represented.

**Figure 8:**
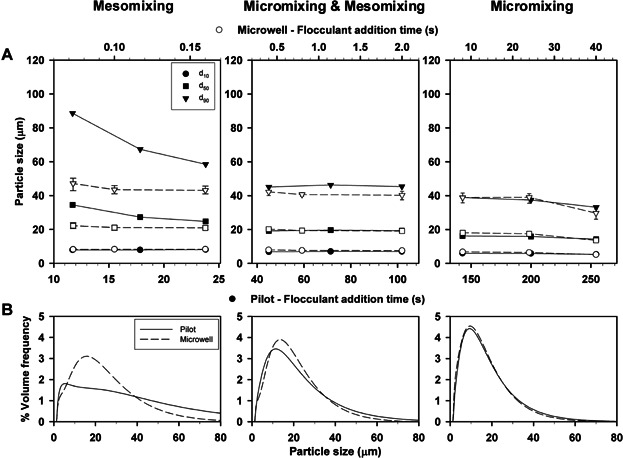
Validation of ε_avg_ as the scale-up basis between the microwell and pilot scale flocculation systems. The graphs describe the effect of mixing time scales on the success of ε_avg_ as the scale-up correlation. ε_avg_ was kept constant at 4.57 × 10^−2^ W.kg^−1^. Error bars represent range of values where n = 8. PSD curves (B) were obtained at 184, 47.6 and 8.6 mL.min^−1^ (pilot) and 900, 50 and 1 µL.s^−1^ (microwell) flocculant addition rates.

Figure 8A shows the USD flocculation system predicted the pilot scale PSD within a ±5% error margin when micromixing was the controlling mixing time scale. For this mixing regime, the PSD curves generated by the two non-geometrically similar systems (Fig. 8B) overlapped each other with their peak volume frequencies between 9 and 10 µm. The influence of mesomixing led to broader PSDs at both flocculation scales. Under the micromixing and mesomixing-controlled regime the values of d_10_, d_50_, and d_90_ for the STR were predicted within a ±11% error margin by the USD system. However, the PSD curves revealed a shift towards larger particles in the USD and STR flocculation systems. In the mesomixing-controlled regime the STR d_10_ data set was predicted within a ±5% error margin. However, the d_50_ and d_90_ STR data sets were almost 50% larger in size than those of the USD system. When compared to smaller mixing times scales, the mesomixing regime led to an increase in the value of d_90_ between 30–170% in the STR and between 5–60% in the USD system. The greater sensitivity of the pilot scale flocculation system to the influence of mesomixing was possible due to a longer lasting effect of the flooding of the STR impeller region by the polymer caused by the fast flocculant addition rates used (see section 4.1).

## Conclusions

This paper describes the key parameters to consider when scaling up a flocculation process between two geometrically different vessels that differ by more than three orders of magnitude in scale. The predominant mixing time scale in the vessel (i.e. micromixing, mesomixing or both) proved to be a key variable affecting the size and complexity of the distribution of the flocs. Impeller speed determined the growth and breakage of the particles thus the final PSD. An USD methodology to mimic the flocculation performance of a pilot scale vessel (1.5 L) of standard configuration using a microliter scale mixing device (800 µL) based on the understanding of the mixing fundamentals is proposed. When a micromixing controlled regime is maintained by using t_add_ > t_crit_, constant ε_avg_ or constant v_tip_ can be used as the scale-up basis. However, constant ε_avg_ should be the scale-up criteria of choice when the distribution of the small particles needs to be tightly correlated, which is typically critical for solid-liquid separations. Using this method the PSD of the larger vessel used was mimicked within a ±5% error margin by the microwell mixing system presented in this paper. Failing to guarantee micromixing as the predominant mixing time scale during scale-up may lead to PSD predictions that differ by up to 50% in size.

## Nomenclature

**Table:** 

Λ_c_	macroscale concentration (m)
T	torque force (N.m)
ε	local turbulent energy dissipation per unit mass (W.kg^−1^)
ε_avg_	average turbulent energy dissipation per unit mass (W.kg^−1^)
ε_max_	maximum energy dissipation per unit mass (W.kg^−1^)
ν	kinematic viscosity (m^2^.s^−1^)
µ	liquid dynamic viscosity (Pa.s)
ρ	liquid density (kg.m^−3^)
A	large eddy disintegration mesomixing constant (dimensionless)
C	off-bottom clearance (m)
D_i_	impeller diameter (m)
D_b_	baffle diameter (m)
D_T_	vessel diameter (m)
D_t_	turbulent diffusivity (m^2^.s^−1^)
d_10_	particle diameter below which 10% of the sample volume exists (µm)
d_50_	particle diameter below which 50% of the sample volume exists (µm)
d_90_	particle diameter below which 90% of the sample volume exists (µm)
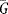	average shear rate (s^−1^)
H_b_	baffle to vessel wall clearance (m)
H_T_	liquid height (m)
M	impeller blade height (m)
N	impeller speed (s^−1^)
P	power input (W)
P_o_	impeller power number (dimensionless)
Q_b_	feed rate (m^3^.s^−1^)
Re	Reynolds number (dimensionless)
t_add_	flocculant or acid addition time (s)
t_c_	circulation time (s)
t_crit_	critical addition time (s)
t_d_	dispersive mesomixing time (s)
t_e_	micromixing time (s)
t_m_	macromixing time (s)
t_s_	large eddy disintegration mesomixing time (s)
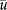	fluid velocity in the vicinity of the feed pipe (m.s^−1^)
V_L_	liquid volume (m^3^)
v_tip_	impeller tip speed (m.s^−1^)
X_S_	segregation index (dimensionless)
Z	distance between impeller blade and feeding point (m)

## Abbreviations

cGMP: current good manufacturing practise
PEI: poly(ethyleneimine)
PSD: particle size distribution
STR: stirred tank reactor
USD: ultra scale-down
